# Joint Forces for Child and Adolescent Psychiatry Training: 1st ESCAP/UEMS-CAP/EFPT Training Day for CAP Trainees

**DOI:** 10.1192/j.eurpsy.2022.2195

**Published:** 2022-09-01

**Authors:** A. Seker, K. Kotsis, P. Deschamps

**Affiliations:** 1 Cambridge and Peterborough NHS Foundation Trust, Child/adolescent Psychiatry, Cambridge, United Kingdom; 2 University of Ioannina, Child/adolescent Psychiatry, Ioannina, Greece; 3 Utrecht University, Child/adolescent Psychiatry, Utrecht, Netherlands

**Keywords:** UEMS-CAP, child and adolescent psychiatry training, ESCAP, EFPT

## Abstract

**Introduction:**

As the Covid-19 pandemic brought about travel and social restrictions, many activities including specialty training events for medical specialty trainees moved online. The European Federation of Psychiatric Trainees, European Society for Child and Adolescent Psychiatry and hild and Adolescent Psychiatry (CAP) Section of UEMS joined forces to turn challenges into an opportunity for CAP trainees and jointly organized the 1st ESCAP/UEMS-CAP/EFPT Training Day.

**Objectives:**

The main aim was to offer CAP trainees throughout Europe high quality and up to date training content free of charge, making use of the different strengths of the organizing associations.

**Methods:**

Content of the Training Day was prepared according to feedback and demand from CAP trainees, collected through a questionnaire prior to the event. This event took place online and ran 9 webinars/workshops as well as a plenary case session where trainees presented real life cases made more challenging with the pandemic and representatives from 3 organizing associations discussed the cases from different perspectives. Remaining webinars/workshops covered a wide range of themes including but not limited to research, leadership, administrative and management skills as well as scientific topics such as eating disorders, medically unexplained symptoms, psychosis.

**Results:**

Almost 200 CAP trainees from 31 countries participated in the event and received certificates of completion. The outcome of the event is being evaluated via quantitative and qualitative methods and similar events will be planned accordingly.

**Conclusions:**

1st ESCAP/UEMS-CAP/EFPT Training Day for CAP trainees was a success for reaching many trainees from across Europe and experimenting with different formats which will inspire future initiatives.

**Disclosure:**

No significant relationships.

